# Supplementary material for: Clinical gait analysis using video-based pose estimation: Multiple perspectives, clinical populations, and measuring change
Source: PLOS Digit Health. 2024 Mar 26;3(3):e0000467. doi: 10.1371/journal.pdig.0000467 (PMC10965062; doi:10.1371/journal.pdig.0000467)
Supplement: S1 Fig — (PDF) [file pdig.0000467.s001.pdf]

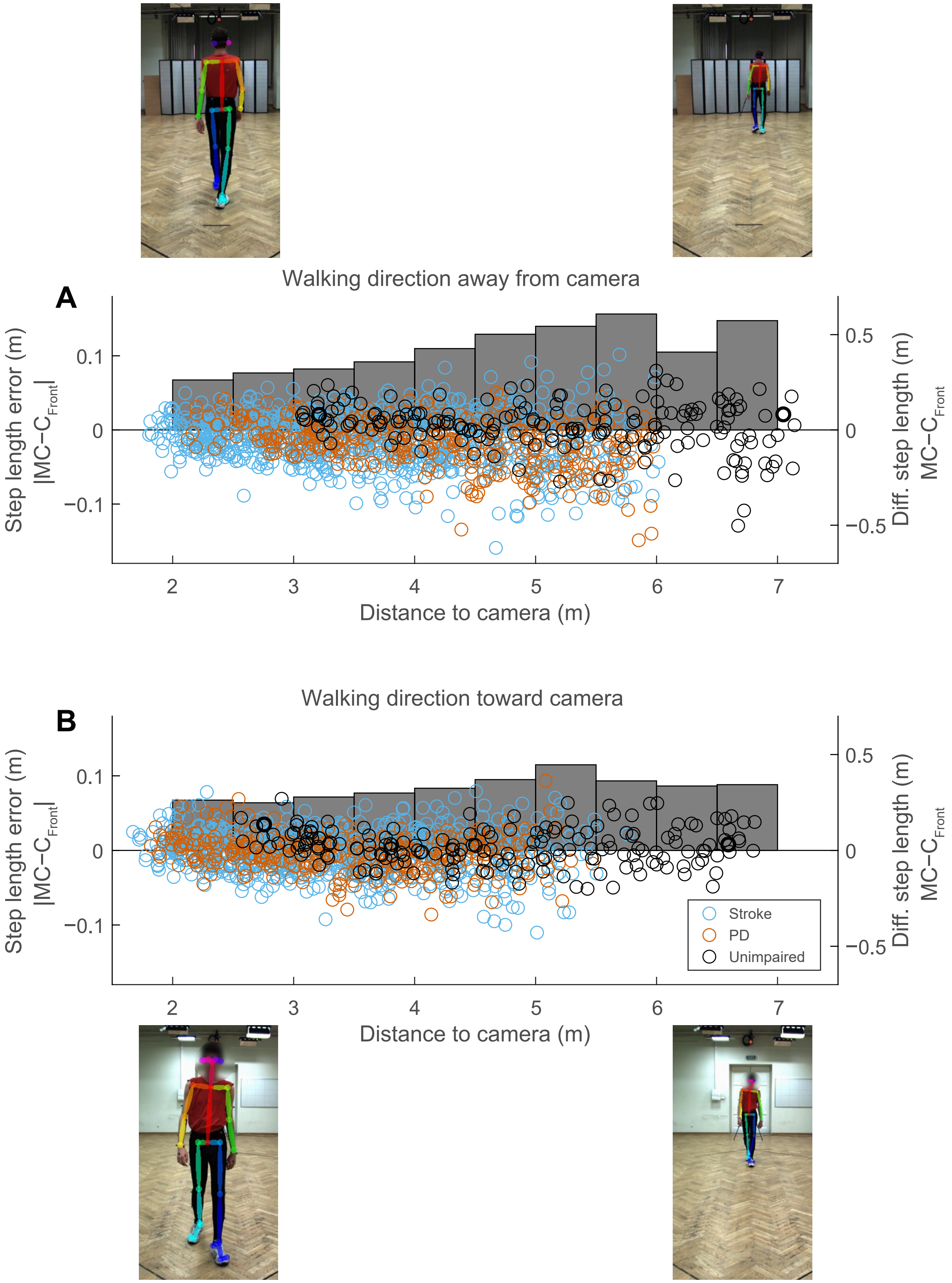

S1 Fig. Step length errors and differences of frontal plane workflow relative to person's distance to camera. Errors and differences relative to distance when the person is walking away from the camera (A) or toward the camera (B). Bar graphs show average step length errors binned across 0.5 m and values are represented on left-hand y-axes. Circles show step length differences for individual steps and values are shown on right-hand y-axes. Image inserts show example frames of a person near to or far away from frontal plane cameras (A, walking away from; B, walking towards).
