# Supplementary material for: Clinical gait analysis using video-based pose estimation: Multiple perspectives, clinical populations, and measuring change
Source: PLOS Digit Health. 2024 Mar 26;3(3):e0000467. doi: 10.1371/journal.pdig.0000467 (PMC10965062; doi:10.1371/journal.pdig.0000467)
Supplement: S2 Fig — (PDF) [file pdig.0000467.s002.pdf]

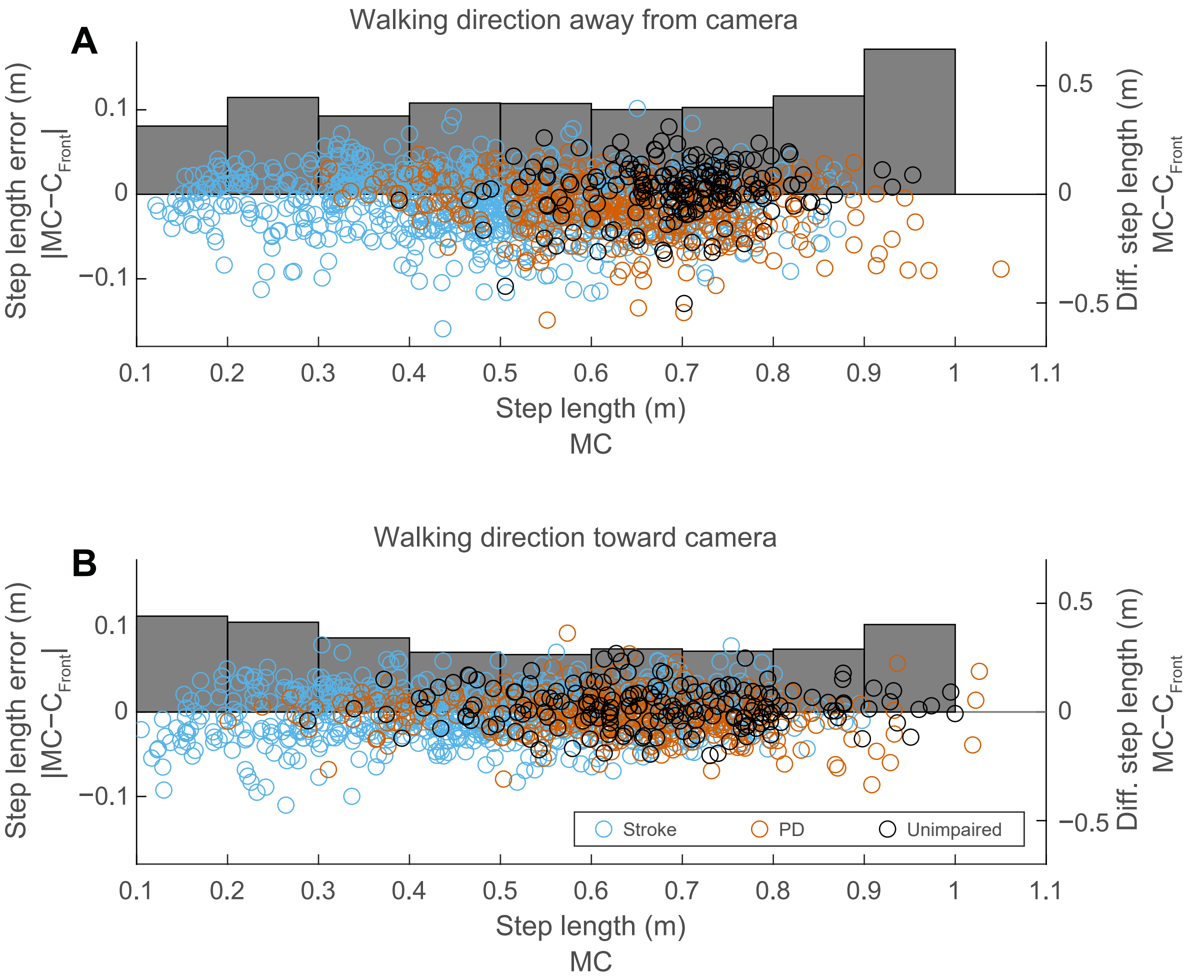

S2 Fig. Step length errors and differences of frontal plane workflow relative to magnitude of step length. Errors and differences relative to step length magnitude when the person is walking away from the camera (A) or toward the camera (B). Bar graphs show average errors binned across 0.1-m intervals and values are represented on left-hand y-axes. Data points show differences for individual steps and values correspond to right-hand y-axes.
