# Supplementary material for: Clinical gait analysis using video-based pose estimation: Multiple perspectives, clinical populations, and measuring change
Source: PLOS Digit Health. 2024 Mar 26;3(3):e0000467. doi: 10.1371/journal.pdig.0000467 (PMC10965062; doi:10.1371/journal.pdig.0000467)
Supplement: S3 Fig — (PDF) [file pdig.0000467.s003.pdf]

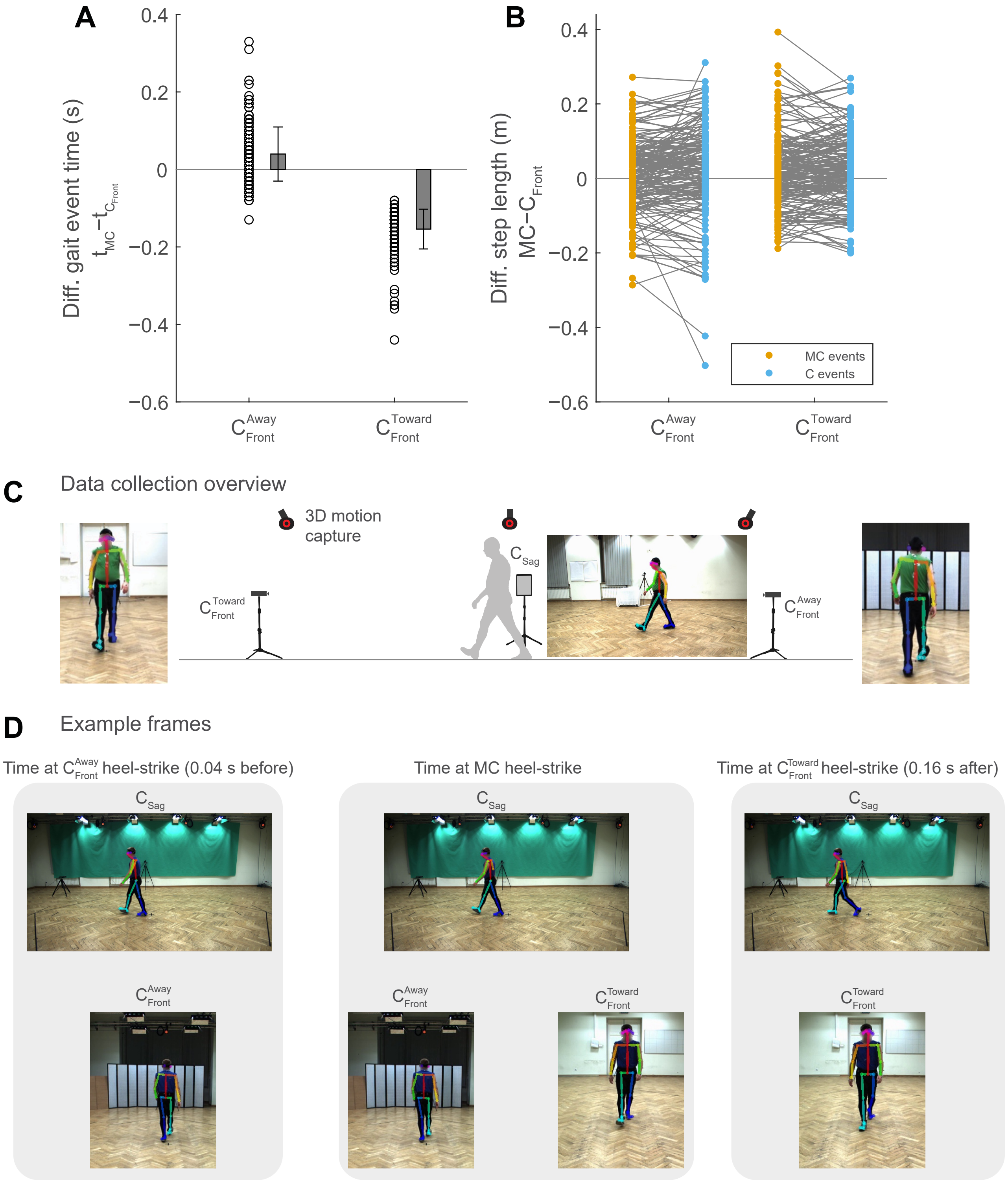

S3 Fig. Influence of gait event timings on step length errors when using frontal plane workflow. Gait events times (heel-strikes) found using the frontal plane workflow are on average detected 0.04 s before the gait events detected from motion capture when the person is walking away from the frontal plane camera, and 0.15 s after motion capture when the person is walking toward the frontal plane camera (A). The effect of differences in gait event times on step length differences is shown in panel B. Data collection overview (C). Example frames of sagittal and frontal camera views of one participant at gait event times detected by the frontal camera that the person walks away from (left), detected by motion capture (middle) and detected by the frontal camera that the person walks toward (right). Note that this analysis can only be performed using the unimpaired data set because recordings of motion capture and video data where synchronized.
