# Supplementary material for: Clinical gait analysis using video-based pose estimation: Multiple perspectives, clinical populations, and measuring change
Source: PLOS Digit Health. 2024 Mar 26;3(3):e0000467. doi: 10.1371/journal.pdig.0000467 (PMC10965062; doi:10.1371/journal.pdig.0000467)
Supplement: S4 Fig — (PDF) [file pdig.0000467.s004.pdf]

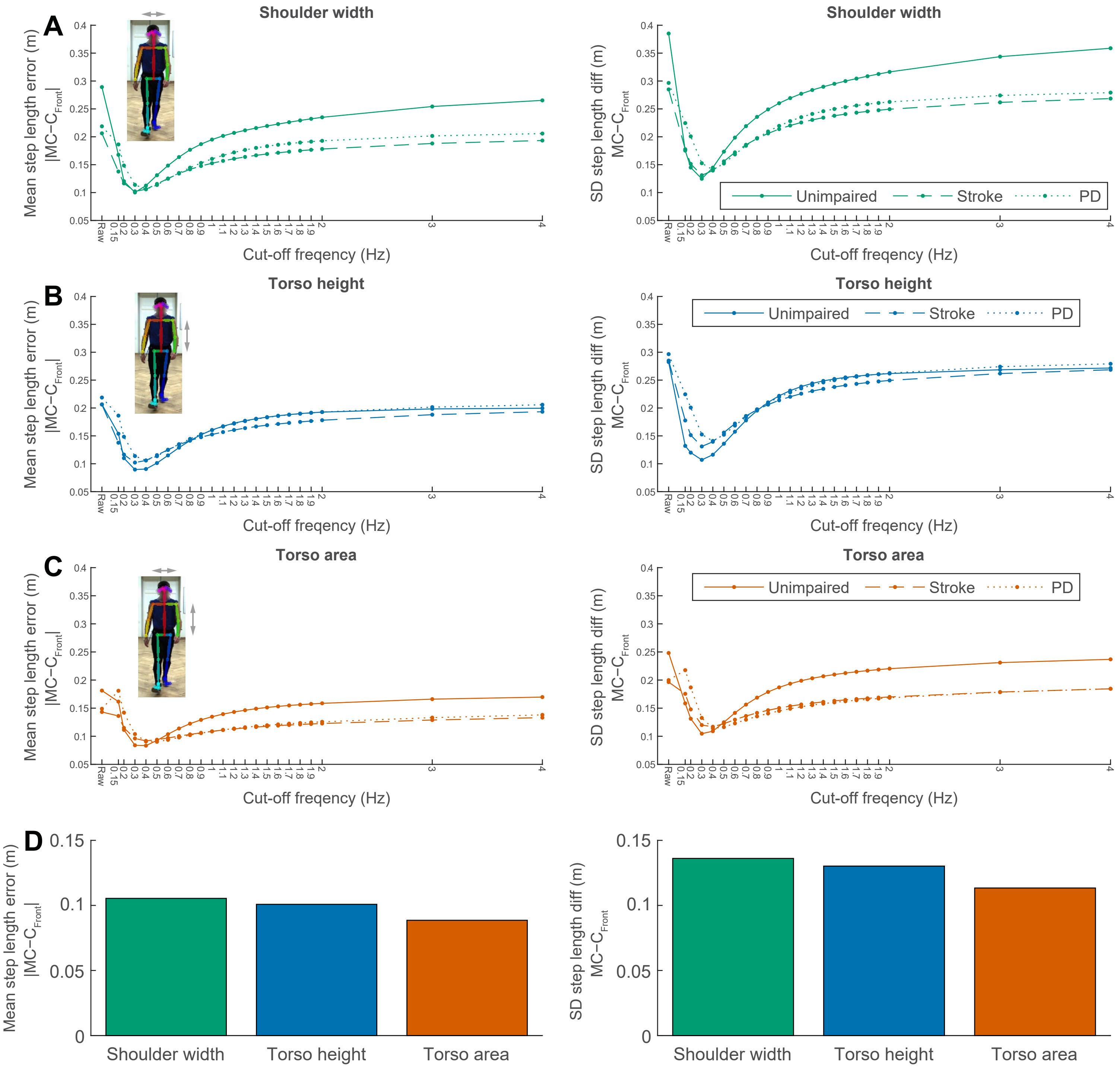

S4 Fig. Evaluation of tracking methods and smoothing using frontal plane workflow. We developed a frontal plane workflow that uses the pixel size of the person to calculate depth-changes throughout the walking bout (see Methods in main text). We evaluated three tracking methods: shoulder width (A), torso height (B) and torso area (C). Shoulder width is the horizontal distance between left and right shoulder keypoints, torso height is the vertical distance between the Neck and MidHip keypoints and torso area is the square root of the product of shoulder width and torso height. Furthermore, we evaluated the best smoothing method of pixel size ratios: we used raw data and low-pass filtered using cut-off frequencies ranging from 0.15 to 0.4 Hz. We calculated the mean step length error (left) and SD of step length differences (right) in order to evaluate the best method. Panel D shows the performance of the tracking methods at the optimal smoothing frequency for each method (averaged across unimpaired, stroke and PD data sets).
