# Supplementary material for: Clinical gait analysis using video-based pose estimation: Multiple perspectives, clinical populations, and measuring change
Source: PLOS Digit Health. 2024 Mar 26;3(3):e0000467. doi: 10.1371/journal.pdig.0000467 (PMC10965062; doi:10.1371/journal.pdig.0000467)
Supplement: S5 Fig — (PDF) [file pdig.0000467.s005.pdf]

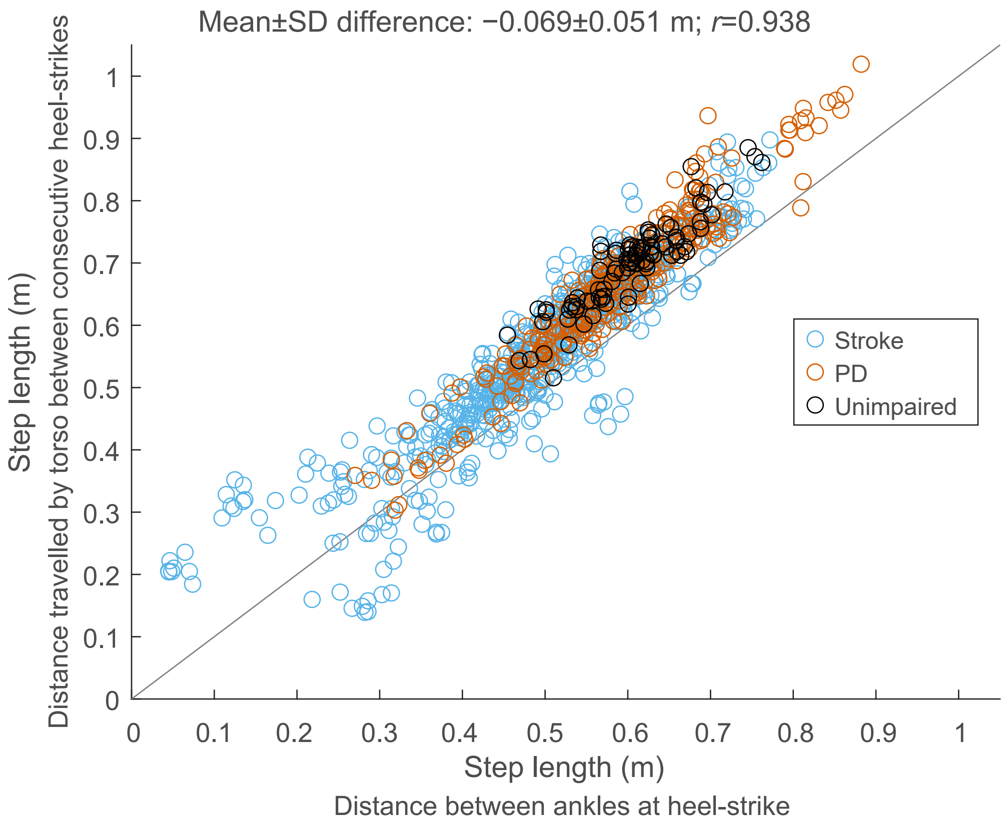

S5 Fig. Comparison of two methods to calculate step length. We used two methods to calculate step lengths; 1) as the horizontal distance between ankle markers or keypoints at instants of heel-strike (x-axis) and 2) as the distance travelled by the torso between consecutive bilateral heel-strikes (y-axis). We used the distance travelled by the torso because the distances between the ankles at a heel-strike instant cannot be obtained from frontal plane videos. When comparing step lengths between motion capture and sagittal plane video we used the distance between the ankles; all step length comparisons with frontal plane data used the distance travelled by the torso.
