# Supplementary material for: Clinical gait analysis using video-based pose estimation: Multiple perspectives, clinical populations, and measuring change
Source: PLOS Digit Health. 2024 Mar 26;3(3):e0000467. doi: 10.1371/journal.pdig.0000467 (PMC10965062; doi:10.1371/journal.pdig.0000467)
Supplement: S1 Table — (PDF) [file pdig.0000467.s006.pdf]

S1 Table Comparison of spatiotemporal gait parameters of the unimpaired group [a]

| Gait Parameter | Difference (Mean±SD) | | | Error (Mean±SD) | | | 95% Limits of Agreement | | |
|---|---|---|---|---|---|---|---|---|---|
| | MC−$C_{F,A}$ | MC−$C_{F,T}$ | $C_{F,A}$−$C_{F,T}$ | \|MC−$C_{F,A}$\| | \|MC−$C_{F,T}$\| | \|$C_{F,A}$−$C_{F,T}$\| | MC−$C_{F,A}$ | MC−$C_{F,T}$ | $C_{F,A}$−$C_{F,T}$ |
| Step time (s) | | | | | | | | | |
| Step | 0.01±0.06 | −0.00±0.05 | −0.02±0.07 | 0.04±0.04 | 0.03±0.04 | 0.05±0.05 | −0.10; 0.13 | −0.10; 0.10 | −0.15; 0.11 |
| Trial | 0.01±0.02 | −0.00±0.02 | −0.02±0.02 | 0.02±0.01 | 0.01±0.01 | 0.02±0.02 | −0.02; 0.05 | −0.03; 0.03 | −0.05; 0.02 |
| Step length (m) | | | | | | | | | |
| Step | 0.020±0.124 | 0.022±0.093 | 0.001±0.156 | 0.094±0.082 | 0.074±0.060 | 0.123±0.095 | −0.222; 0.263 | −0.160; 0.203 | −0.305; 0.307 |
| Trial | 0.020±0.032 | 0.021±0.037 | 0.001±0.031 | 0.029±0.025 | 0.034±0.025 | 0.024±0.019 | −0.043; 0.084 | −0.052; 0.094 | −0.060; 0.062 |
| Gait speed (m $s^{-1}$) | | | | | | | | | |
| Trial | 0.01±0.06 | 0.04±0.06 | 0.04±0.06 | 0.05±0.04 | 0.06±0.05 | 0.06±0.04 | −0.11; 0.12 | −0.08; 0.17 | −0.09; 0.16 |

MC, motion capture; $C_{F,A}$, frontal plane camera that person walks away from; $C_{F,T}$, frontal plane camera that person walks toward

[a] Values of spatiotemporal gait parameters calculated as trial-level averages and for individual steps.
