# Supplementary material for: Clinical gait analysis using video-based pose estimation: Multiple perspectives, clinical populations, and measuring change
Source: PLOS Digit Health. 2024 Mar 26;3(3):e0000467. doi: 10.1371/journal.pdig.0000467 (PMC10965062; doi:10.1371/journal.pdig.0000467)
Supplement: S2 Table — (PDF) [file pdig.0000467.s007.pdf]

S2 Table Spatiotemporal gait parameters for stroke and PD groups [a]

Gait parameter	Preferred speed (Mean±SD)			Fast speed (Mean±SD)		
	MC	C_S	C_F	MC	C_S	C_F
Stroke						
Step time (s)						
Paretic	0.77±0.31	0.76±0.31	0.74±0.32	0.66±0.29	0.65±0.29	0.64±0.28
Non-paretic	0.60±0.07	0.61±0.07	0.61±0.07	0.52±0.07	0.53±0.07	0.53±0.08
Step $\text{length}_{\text{Ankle}}$ (m) [b]						
Paretic	0.480±0.094	0.471±0.100	…	0.550±0.106	0.538±0.113	…
Non-paretic	0.447±0.134	0.438±0.146	…	0.513±0.151	0.501±0.159	…
Step $\text{length}_{\text{Torso}}$ (m) [c]						
Paretic	0.536±0.126	0.522±0.125	0.579±0.111	0.610±0.138	0.599±0.142	0.665±0.135
Non-paretic	0.510±0.130	0.485±0.142	0.527±0.168	0.581±0.147	0.564±0.160	0.600±0.182
Gait $\text{speed}_{\text{Ankle}}$ ($m\ s^{-1}$) [b]						
Trial	0.72±0.24	0.71±0.25	…	0.98±0.35	0.95±0.35	…
Gait $\text{speed}_{\text{Torso}}$ ($m\ s^{-1}$) [c]						
Trial	0.82±0.28	0.79±0.29	0.88±0.30	1.09±0.38	1.07±0.39	1.17±0.42
Step time asym.						
Trial	0.09±0.13	0.08±0.12	0.07±0.13	0.09±0.12	0.07±0.11	0.07±0.11
Step $\text{length}_{\text{Ankle}}$ asym. [b]						
Trial	0.055±0.147	0.058±0.159	…	0.055±0.153	0.057±0.154	…
Step $\text{length}_{\text{Torso}}$ asym. [c]						
Trial	0.025±0.080	0.046±0.068	0.067±0.130	0.025±0.077	0.038±0.055	0.066±0.113
Parkinson's disease						
Step time (s)						
Right	0.56±0.06	0.56±0.06	0.55±0.06	0.47±0.06	0.47±0.06	0.46±0.06
Left	0.56±0.07	0.57±0.06	0.55±0.06	0.47±0.06	0.47±0.06	0.46±0.06
Step $\text{length}_{\text{Ankle}}$ (m) [b]						
Right	0.527±0.094	0.548±0.087	…	0.627±0.090	0.643±0.081	…
Left	0.536±0.107	0.539±0.111	…	0.639±0.089	0.641±0.088	…
Step $\text{length}_{\text{Torso}}$ (m) [c]						
Right	0.607±0.120	0.601±0.122	0.647±0.135	0.721±0.107	0.735±0.108	0.773±0.127
Left	0.603±0.119	0.620±0.123	0.640±0.138	0.715±0.104	0.734±0.112	0.792±0.162
Gait $\text{speed}_{\text{Ankle}}$ ($m\ s^{-1}$) [b]						
Trial	0.95±0.19	0.97±0.19	…	1.36±0.19	1.38±0.19	…
Gait $\text{speed}_{\text{Torso}}$ ($m\ s^{-1}$) [c]						
Trial	1.09±0.22	1.10±0.23	1.18±0.25	1.54±0.22	1.57±0.24	1.70±0.27
Trunk incl. (°) [d]						
Trial	74.5±5.3	74.5±5.0	…	72.0±5.7	72.0±5.0	…

MC, motion capture; C_S, sagittal plane camera; C_F, frontal plane camera

[a] Values of spatiotemporal gait parameters are calculated as session-level averages.

[b] Parameter depending on step length in which step length is calculated as distance between ankles at heel-strike; missing values because step length calculated as ankle-distance cannot be calculated from C_F.

[c] Parameter depending on step length in which step length is calculated as distance travelled by torso between consecutive heel-strikes.

[d] Missing values because trunk inclination cannot be calculated from C_F.
