# Supplementary material for: Clinical gait analysis using video-based pose estimation: Multiple perspectives, clinical populations, and measuring change
Source: PLOS Digit Health. 2024 Mar 26;3(3):e0000467. doi: 10.1371/journal.pdig.0000467 (PMC10965062; doi:10.1371/journal.pdig.0000467)
Supplement: S3 Table — (PDF) [file pdig.0000467.s008.pdf]

S3 Table Comparison of spatiotemporal gait parameters of stroke and PD groups calculated as trial averages

Gait Parameter	Difference (Mean±SD)			Error (Mean±SD)			95% Limits of Agreement		
	$MC-C_S$	$MC-C_F$	C_F-C_S	$\lvert MC-C_S \rvert$	$\lvert MC-C_F \rvert$	$\lvert C_S-C_F \rvert$	$MC-C_S$	$MC-C_F$	C_F-C_S
Stroke									
Step time (s)									
Away from C_F [a]	0.00±0.02	0.02±0.07	0.02±0.07	0.02±0.01	0.05±0.05	0.05±0.05	−0.04; 0.04	−0.12; 0.16	−0.12; 0.16
Toward C_F	0.00±0.03	−0.01±0.09	−0.01±0.09	0.02±0.02	0.06±0.07	0.05±0.07	−0.06; 0.06	−0.18; 0.17	−0.17; 0.16
Step length (m) [b]									
Away from C_F	0.011±0.036	−0.056±0.090	−0.072±0.092	0.028±0.026	0.084±0.064	0.091±0.073	−0.059; 0.082	−0.233; 0.120	−0.252; 0.108
Toward C_F	0.009±0.042	−0.013±0.077	−0.029±0.082	0.030±0.031	0.062±0.048	0.069±0.053	−0.074; 0.092	−0.165; 0.139	−0.190; 0.132
Gait speed ($m\ s^{-1}$) [b]									
Away from C_F	0.02±0.05	−0.13±0.11	−0.15±0.09	0.04±0.04	0.14±0.10	0.15±0.09	−0.09; 0.12	−0.34; 0.08	−0.33; 0.03
Toward C_F	0.02±0.09	−0.01±0.09	−0.04±0.10	0.04±0.07	0.06±0.06	0.08±0.08	−0.15; 0.19	−0.18; 0.16	−0.24; 0.17
Step time asym.									
Away from C_F	0.01±0.03	−0.00±0.09	−0.01±0.09	0.02±0.02	0.07±0.06	0.07±0.06	−0.05; 0.07	−0.19; 0.18	−0.19; 0.16
Toward C_F	0.02±0.04	0.04±0.10	0.03±0.09	0.03±0.03	0.08±0.08	0.07±0.07	−0.06; 0.09	−0.15; 0.24	−0.15; 0.21
Step length asym. [b]									
Away from C_F	−0.003±0.068	−0.064±0.143	−0.041±0.144	0.047±0.049	0.113±0.109	0.107±0.103	−0.136; 0.130	−0.345; 0.216	−0.322; 0.241
Toward C_F	−0.001±0.088	−0.021±0.165	−0.010±0.134	0.055±0.069	0.103±0.130	0.095±0.095	−0.174; 0.172	−0.344; 0.303	−0.273; 0.252
Parkinson's disease									
Step time (s)									
Away from C_F	−0.00±0.01	0.02±0.04	0.02±0.04	0.01±0.01	0.03±0.03	0.03±0.03	−0.03; 0.03	−0.05; 0.09	−0.06; 0.10
Toward C_F	−0.00±0.02	0.00±0.03	0.00±0.03	0.01±0.01	0.02±0.02	0.02±0.02	−0.03; 0.03	−0.06; 0.06	−0.06; 0.06
Step length (m) [b]									
Away from C_F	−0.004±0.021	−0.082±0.086	−0.071±0.091	0.018±0.012	0.092±0.074	0.091±0.070	−0.046; 0.038	−0.249; 0.086	−0.249; 0.106
Toward C_F	−0.017±0.024	−0.021±0.070	−0.009±0.072	0.025±0.017	0.055±0.048	0.058±0.044	−0.065; 0.030	−0.158; 0.117	−0.151; 0.132
Gait speed ($m\ s^{-1}$) [b]									
Away from C_F	−0.01±0.04	−0.21±0.15	−0.19±0.15	0.03±0.02	0.23±0.13	0.22±0.12	−0.08; 0.07	−0.52; 0.09	−0.50; 0.11
Toward C_F	−0.03±0.04	−0.03±0.09	−0.01±0.11	0.04±0.03	0.08±0.06	0.08±0.07	−0.10; 0.04	−0.22; 0.15	−0.22; 0.20
Trunk incl. (°) [c]									
Away from C_F	−0.6±2.0	…	…	1.6±1.2	…	…	−4.4; 3.3	…	…
Toward C_F	0.6±1.4	…	…	1.2±0.8	…	…	−2.2; 3.3	…	…

MC, motion capture; C_S, sagittal plane camera; C_F, frontal plane camera

[a] Values are shown for separate walking directions: 1) trials in which the person walks away from C_F with their left side turned to C_S or 2) trials where the person walks toward C_F with their right side turned to C_S.

[b] Parameter depending on step length: comparisons of MC and C_S, step length calculated as distance between ankles at heel-strike; comparisons of MC and C_F and of C_S and C_F, step length calculated as distance travelled by torso between consecutive heel-strikes.

[c] Missing values because trunk inclination cannot be calculated from C_F.
