# Supplementary material for: Clinical gait analysis using video-based pose estimation: Multiple perspectives, clinical populations, and measuring change
Source: PLOS Digit Health. 2024 Mar 26;3(3):e0000467. doi: 10.1371/journal.pdig.0000467 (PMC10965062; doi:10.1371/journal.pdig.0000467)
Supplement: S4 Table — (PDF) [file pdig.0000467.s009.pdf]

S4 Table Comparison of spatiotemporal gait parameters of stroke and PD groups calculated for individual steps

Gait Parameter	Difference (Mean±SD)			Error (Mean±SD)			95% Limits of Agreement		
	$MC-C_S$	$MC-C_F$	C_F-C_S	$\lvert MC-C_S\rvert$	$\lvert MC-C_F\rvert$	$\lvert C_S-C_F\rvert$	$MC-C_S$	$MC-C_F$	C_F-C_S
Stroke									
Step time (s)									
Away from C_F [a]	0.00±0.04	0.02±0.12	0.02±0.12	0.03±0.02	0.09±0.08	0.09±0.08	−0.07; 0.07	−0.21; 0.24	−0.21; 0.24
Toward C_F	0.00±0.04	−0.01±0.13	−0.01±0.13	0.03±0.03	0.08±0.10	0.08±0.10	−0.08; 0.08	−0.27; 0.25	−0.27; 0.25
Step length (m) [b]									
Away from C_F	0.016±0.058	−0.051±0.125	−0.067±0.128	0.046±0.039	0.104±0.086	0.109±0.096	−0.096; 0.129	−0.296; 0.194	−0.318; 0.184
Toward C_F	0.013±0.057	−0.012±0.103	−0.025±0.103	0.045±0.036	0.080±0.066	0.083±0.066	−0.098; 0.124	−0.214; 0.190	−0.227; 0.176
Parkinson's disease									
Step time (s)									
Away from C_F	−0.00±0.02	0.02±0.06	0.02±0.06	0.02±0.01	0.05±0.04	0.05±0.05	−0.04; 0.04	−0.10; 0.14	−0.11; 0.15
Toward C_F	−0.00±0.02	0.00±0.04	0.00±0.05	0.02±0.01	0.03±0.03	0.04±0.03	−0.05; 0.04	−0.09; 0.09	−0.09; 0.10
Step length (m) [b]									
Away from C_F	−0.009±0.042	−0.070±0.125	−0.062±0.131	0.035±0.026	0.107±0.095	0.109±0.095	−0.092; 0.074	−0.315; 0.174	−0.318; 0.195
Toward C_F	−0.010±0.041	−0.016±0.094	−0.006±0.102	0.033±0.026	0.072±0.062	0.078±0.065	−0.090; 0.070	−0.200; 0.167	−0.206; 0.193

MC, motion capture; C_S, sagittal plane camera; C_F, frontal plane camera

[a] Values are shown for separate walking directions: 1) trials in which the person walks away from C_F with their left side turned to C_S or 2) trials where the person walks toward C_F with their right side turned to C_S.

[b] Parameter depending on step length: comparisons of MC and C_S, step length calculated as distance between ankles at heel-strike; comparisons of MC and C_F and of C_S and C_F, step length calculated as distance travelled by torso between consecutive heel-strikes.
