# Supplementary material for: Clinical gait analysis using video-based pose estimation: Multiple perspectives, clinical populations, and measuring change
Source: PLOS Digit Health. 2024 Mar 26;3(3):e0000467. doi: 10.1371/journal.pdig.0000467 (PMC10965062; doi:10.1371/journal.pdig.0000467)
Supplement: Attachment — Submitted filename: Response to Reviewers 7.24.2023.pdf [file pdig.0000467.s010.pdf]

Re: PDIG-D-23-00173 "CLINICAL GAIT ANALYSIS USING VIDEO-BASED POSE ESTIMATION: MULTIPLE PERSPECTIVES, CLINICAL POPULATIONS, AND MEASURING CHANGE"

Reviewer 1

The authors present a simple and low-cost quantitative gait analysis system for clinical applications.

Although the intent is very commendable, in my opinion the results do not support the clinical usability of the system.

These are the main reasons:

- in comparison with the literature, it is not enough to talk about the costs and simplicity of the protocol and its implementation, but it is necessary to carefully evaluate and compare the reliability, accuracy and repeatability of the system performance, which does not appear in this manuscript.

We thank the reviewer for their time and comments and we recognize the important point brought up here. We agree that any statements about cost and simplicity should not be regarded without concern for the workflows' performance compared to gold standard measurement systems. Our manuscript contains a thorough evaluation of the accuracy of our video-based workflows as compared to a marker-based 3D motion capture system (see Figs. 2 to 6, Tables 1 and 2). We provide context to the results in the Discussion (page 15 to 16, lines 343-355):

"*It is informative to consider the accuracy of our workflow relative to reported test-retest minimal detectable change or minimal clinical important difference values of the population of interest. For example, a meaningful change in gait speed is often reported as 0.10 m s^{-1}[20], but may vary from 0.05 up to 0.30 m s^{-1} depending on the population studied[21–32]. The average errors of our video-based measurements relative to motion capture generally fall within these margins, suggesting that gait speed is likely to be reliably measured in many populations (e.g., older adults, post-stroke, PD, following hip fracture, cerebral palsy, multiple sclerosis) using our workflow. Minimal detectable changes in gait kinematics may also be dependent on the population of interest, with estimates ranging from about 4° to 11° of lower-limb sagittal plane kinematics[26,28,33–36]. Average errors of sagittal plane hip and knee kinematics in our study were*

less than 4°, while errors at the ankle were up to 6.8°, suggesting that hip and knee kinematics from our workflow can be accurately tracked while continued improvement in measurement of ankle angles is needed."

- methodologically, the control group must perform the same protocol as the experimental group, with the same experimental apparatus and setting. The same goes for data pre-processing.

The unimpaired, stroke and PD groups all performed walking bouts across a walkway. Note that we have performed the sagittal plan analysis in the healthy group and presented those data in a previous paper (Stenum et al. (2021), PLOS Computation Biology). As such, we only present the frontal plane workflow in the unimpaired group in this manuscript. We consider it a strength of the manuscript that the control group was collected outside of our lab and that we can obtain accurate gait parameters with these different datasets collected outside of our laboratory.

- in data processing, corrections have been made by visual inspection of the data (not automatic), which is typically time-consuming. An automated processing should be implemented, with a view to having a truly simple and easily usable system in the clinic, as stated by the authors.

We agree that automation of the workflow is an important concern. We are actively pursuing automation of our workflow in order to increase its utility. On the other hand, we feel that there is a benefit to presenting this work in its current state. This allows other researchers to build, customize and automate the workflow for their applications.

- in the presentation of the methods, some offsets were found between the reference instrumentation and the one tested by the authors; the offset has been removed in the processing, but there is no exhaustive justification for the presence of such an offset.

We found an offset of 12° of trunk inclination between motion capture and sagittal plane video. Trunk inclination was calculated as the angle relative to vertical between the mid-hip and neck keypoints at heel-strikes in the sagittal plane videos and the angle between the C7 and right PSIS markers at heel-strikes in the motion capture data. There are correspondences between the mid-hip keypoint and PSIS marker and between the neck keypoint and C7 marker. However, the keypoints and markers do not exactly align and as a result there is a consistent bias (offset) of trunk inclination when calculated using video data and marker-based 3D motion capture. A primary reason for this offset is that the neck keypoint is identified anterior relative to the

position of the C7 marker. The graph below shows the comparison between trunk inclination when calculated from motion capture a sagittal plane video without the offset. There is a high correlation between the methods (r^2=0.929) and the average difference is 12°.

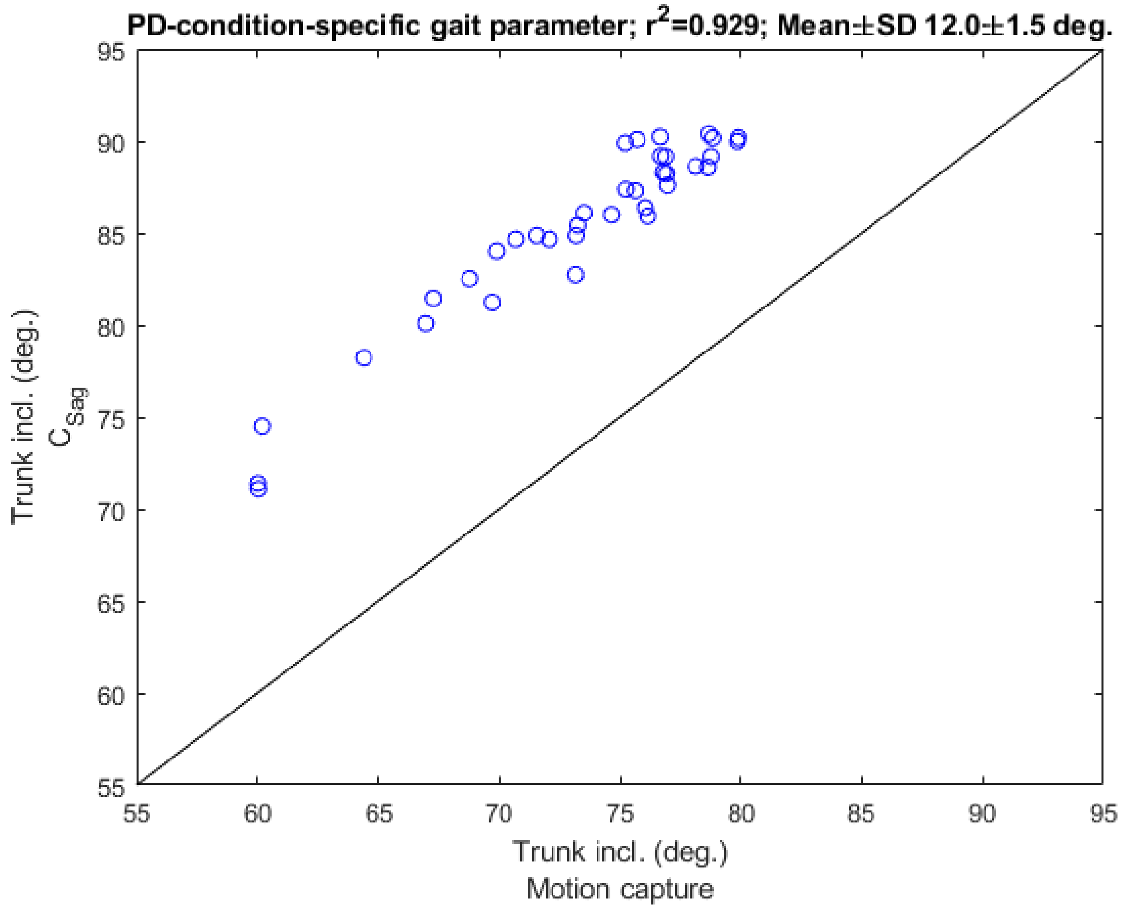

We have added (highlighted in red) the following to the description of the trunk inclination offset (page 26; lines 589 to 594): *"During initial comparisons we found an offset (mean±SD 12.0°±1.5°) between motion capture and sagittal plane video data; we subtracted a fixed offset of 12° from trunk inclination in the sagittal plane video data in order to create a better numeric comparison with the motion capture data. The offset is a consequence of the fact that video-based keypoints and markers track similar anatomical regions, but do not track the* exact *same anatomical locations."*

Reviewer 2

Appreciate if the authors can mention what is the level of agreement with standard marker-based motion capture systems, instrumented gait mats.

We thank the reviewer for their time. We have presented levels of agreement with marker-based 3D motion capture in Tables 1 and 2 and Supplemental Tables S1, S3 and S4.

This is a well written article with simple explanation.

We thank the reviewer for their kind remarks.

Reviewer 3

In this manuscript, the authors demonstrated a valuable study to perform clinical relevant gait analysis with simple videos from two tablets cameras. The background and advances of this study were very clearly reported and so were the experimental design and results. Thus, I recommend acceptance of this work after minor revision based on the following comments.

We thank the reviewer for their time and expertise.

In Fig.1C, there is a major difference of adding or not adding low-pass filter in the sagittal workflow and frontal workflow, what is the reason of this difference?

We acknowledge that Fig. 1C only states a low-pass filter step for the sagittal workflow. However, the ‘Calculate depth-change time-series’ step in the frontal workflow also contains a low-pass filter step explained in the Methods (pages 23 to 24, lines 525 to 538) and Fig. S4. Briefly, the size ratio time-series (calculated as torso area divided by initial torso area) was low-pass filtered using a cut-off frequency of 0.4 Hz. When presenting an outline of the sagittal and frontal workflows we chose the heading ‘Calculate depth-change time-series’ as a simplified means of describing this step.

We have added the following (highlighted in red) to the figure legend concerning panel C (page 34, lines 751 to 756): *“We developed workflows to perform a gait analysis, independently, for videos of the frontal and sagittal plane (C). See Methods section for detailed information about the frontal and sagittal plane post-processing workflows. Note that the ‘Calculate depth-change time-series’ step in the frontal workflow contains multiple sub-steps including tracking the pixel size of the torso and low-pass filtering (see Fig. S4 for justification of tracking method and smoothing).”*

The authors mentioned that the unimpaired study data was from a public dataset, which has a different camera setup with the one used in the study. Is there a specific

consideration on utilizing the public dataset instead of collecting user data under the same front + sagittal camera setup?

In the current manuscript we have only presented the frontal videos of the unimpaired study data – in fact, the unimpaired study contains additional sagittal plane data that we have used previously to develop our sagittal plane workflow (see Stenum et al. (2021), PLOS Computational Biology). Because the sagittal plane data have already been published, we chose to only present the frontal plane video analysis in the current study. Additionally, we consider it a strength of the manuscript that the unimpaired group was collected outside of our lab and that we can obtain accurate gait parameters with these data.
