# Supplementary material for: Clinical gait analysis using video-based pose estimation: Multiple perspectives, clinical populations, and measuring change
Source: PLOS Digit Health. 2024 Mar 26;3(3):e0000467. doi: 10.1371/journal.pdig.0000467 (PMC10965062; doi:10.1371/journal.pdig.0000467)
Supplement: Attachment — Submitted filename: Response to Reviewers.docx [file pdig.0000467.s011.docx]

**Re: PDIG-D-23-00173R1 “Clinical gait analysis using video-based pose estimation: multiple perspectives, clinical populations, and measuring change”**

**Reviewer 1**

**I thank the authors for the opportunity to read this work. I was not involved in the first round of revisions, and as such, do not have any major concerns after reading the revised manuscript and the response to reviewers. Below are a few relatively minor issues that the authors might consider when finalizing the paper for publication.**

We thank the reviewer for their time and comments.

**Introduction, l47: What does the term “data-limited” mean?**

We have used the term “data-limited” to communicate that some technologies only provide predefined parameters (e.g., gait mats only provide spatiotemporal data, wearables might only provide step counts) whereas you can get full-body kinematics with pose with potential to calculate many different parameters.

For clarity we have omitted “data-limited” and changed the sentence to “Other commercially available technologies (e.g., gait mats, wearable systems) only provide redefined parameters (e.g., spatiotemporal data or step counts) ), relatively costly, and require specific hardware”.

**l72: I suggest clarifying that the ‘changes in gait’ referred to here specifically relate to changes that occur in response to a change in speed. The reader might otherwise infer that this refers to changes over the course of a longer-term intervention.**

We have changed the sentence according to the reviewer’s suggestion so that it now reads “the ability to measure changes in gait that occur in response to a change in speed”.

**l80 (starting “We demonstrate”): This is possibly a style issue, but in my view this part of the introduction actually belongs more in the results or discussion section.**

We have omitted this section as suggested by the reviewer. This also helps to minimize repetitive text in the manuscript.

**l83 (also l307): It seems odd to refer to tablets as “household video recording devices” or “common household devices”. Why not low-cost cameras or something similar?**

We have changed “household” to “low-cost” in lines 24, 34, 51, 83, 89 and 307.

**Results, l92-103: This is basically a restating of the aims and methods. No actual results are presented in this section. Is it needed?**

We acknowledge that this section has repetitions that we initially included to give an overview of the methods since the Methods section is at the end of the manuscript. We have omitted the section and moved parts to the introduction (lines 76 to 79; in order to introduce the concept of our novel frontal-plane workflow) and the results (lines 84 to 90; in order to provide a brief overview of the methodology leading to the first set of results in the study).

**l119-127 and 128-138: Here again there is a lot of repetition from the methods, with some reference to figures/tables. I wonder if the text could be streamlined somehow to minimize this repetition?**

Again, we acknowledge that these sections include repetitions. However, we feel that these sections orient the reader to methodological points that are important for context and would otherwise only be apparent in the Methods section at the end of the manuscript.

**l312-3: I think this sentence is misleading because the kind of changes that occur during recovery or rehabilitation do so over a much longer time course than what was measured here, which was a change in speed within a single session. Rehab-induced changes might also be much more subtle, e.g. the change in preferred speed over a 3-month period. I suggest rewording the sentence to something like: “4) that measure within-participant changes in gait as a result of changes in walking speed”.**

This is a good point and it is correct that changes over time may be more subtle than the within-session gait speed changes that we used in this study. We have changed the sentence according to the reviewer’s suggestion so that it now reads “that measure within-participant changes in gait as a result of changes in walking speed”.

**l31-23: This sentence largely repeats the previous paragraph.**

We are unsure which line numbers the reviewer is referencing here. As such, we have not made changes to the manuscript.

**l593-4: This is a good point. Reasons for this are expounded in (at least) the following papers:**

**- https://www.sciencedirect.com/science/article/pii/S0021929021002402**

**-** [**https://nam02.safelinks.protection.outlook.com/?url=https%3A%2F%2Farxiv.org%2Fpdf%2F1907.10226.pdf&data=05%7C01%7Cjstenum1%40jhmi.edu%7C837015674b174596223508dbe20d28dd%7C9fa4f438b1e6473b803f86f8aedf0dec%7C0%7C0%7C638352318672462161%7CUnknown%7CTWFpbGZsb3d8eyJWIjoiMC4wLjAwMDAiLCJQIjoiV2luMzIiLCJBTiI6Ik1haWwiLCJXVCI6Mn0%3D%7C3000%7C%7C%7C&sdata=L8Ft0noEPr7gQh7mXe8E2ssE4dIeukJMjI1%2FisQBuuA%3D&reserved=0**](https://nam02.safelinks.protection.outlook.com/?url=https%3A%2F%2Farxiv.org%2Fpdf%2F1907.10226.pdf&data=05%7C01%7Cjstenum1%40jhmi.edu%7C837015674b174596223508dbe20d28dd%7C9fa4f438b1e6473b803f86f8aedf0dec%7C0%7C0%7C638352318672462161%7CUnknown%7CTWFpbGZsb3d8eyJWIjoiMC4wLjAwMDAiLCJQIjoiV2luMzIiLCJBTiI6Ik1haWwiLCJXVCI6Mn0%3D%7C3000%7C%7C%7C&sdata=L8Ft0noEPr7gQh7mXe8E2ssE4dIeukJMjI1%2FisQBuuA%3D&reserved=0)

We have cited the two suggested and excellent papers for further reading on this issue and pose estimation in general.
